# The adapter protein ADAP is required for selected dendritic cell functions

**DOI:** 10.1186/1478-811X-10-14

**Published:** 2012-06-06

**Authors:** Mauro Togni, Swen Engelmann, Dirk Reinhold, Burkhart Schraven, Annegret Reinhold

**Affiliations:** 1 Institute for Molecular and Clinical Immunology, Otto von Guericke University Magdeburg, Leipziger Strasse 44, 39120 Magdeburg, Germany; 2 Department of Immune Control, Helmoltz Center for Infection Research, Inhoffenstraße 7, 38124 Braunschweig, Germany

**Keywords:** Adapter protein, ADAP, Dendritic cell, Integrin, Inside-out signaling

## Abstract

**Background:**

The cytosolic adaptor protein ADAP (adhesion and degranulation promoting adapter protein) is expressed by T cells, natural killer cells, myeloid cells and platelets. ADAP is involved in T-cell-receptor-mediated inside-out signaling, which leads to integrin activation, adhesion and reorganization of the actin cytoskeleton. However, little is known about the role of ADAP in myeloid cells. In the present study, we analyzed the function of ADAP in bone-marrow-derived dendritic cells (BMDCs) from ADAP-deficient mice.

**Results:**

ADAP-deficient BMDCs showed almost normal levels of antigen uptake, adhesion, maturation, migration from the periphery to the draining lymph nodes, antigen-specific T-cell activation, and production of the proinflammatory cytokines IL-6 and TNF-∝. Furthermore, we provide evidence that the activation of signaling pathways after lipopolysaccharide (LPS) stimulation are not affected by the loss of ADAP. In contrast, ADAP-deficient BMDCs showed defects in CD11c-mediated cellular responses, with significantly diminished production of IL-6, TNF-∝ and IL-10. Actin polymerization was enhanced after CD11c integrin stimulation.

**Conclusions:**

In summary, we propose that the adapter molecule ADAP is critical for selected CD11c integrin-mediated functions of dendritic cells.

## Lay Abstract

Adapter molecules mediate protein-protein interactions in signal transduction cascades. These signaling cascades translate information from cell surface receptors into cellular responses. We focused our research on the adapter molecule ADAP (adhesion and degranulation promoting adapter protein). To investigate the function of ADAP in immune cells we used a genetically engineered mouse lacking this molecule. It is known that ADAP plays a role in integrin-mediated signaling pathways leading to adhesion and motility in T lymphocytes. However, little is known about the role of ADAP in dendritic cells, a special cell population within the immune system linking the innate and the adaptive immunity. Using their long dendrites, these cells capture and process antigen material and present it to other immune cells. Here, we provide evidence that most dendritic cell functions are not affected by the lack of ADAP. Interestingly, ADAP-deficient dendritic cells showed defects in integrin-mediated cellular responses. These findings have important implications for the understanding of the role of ADAP in integrin-mediated signaling cascades in dendritic cells. This knowledge would also facilitate the therapeutic modulation of signal transduction pathways in these cells.

## Introduction

Adaptor proteins play a crucial role in organizing signalosomes, which are molecular complexes involved in signal transduction. Adaptor proteins are subdivided into membrane-anchored adaptor molecules (transmembrane adaptor molecules) and cytosolic adaptor molecules [1]. ADAP (adhesion and degranulation promoting adaptor protein, previously designated SLAP-130 or Fyb) is a cytosolic adaptor molecule expressed by T cells, natural killer (NK) cells, myeloid cells and platelets [2,3]. ADAP is expressed during the early stages of B cell development in the bone marrow, but not in mature B cells [4]. On the structural level, ADAP consists of a unique N-terminal region, a proline-rich region, multiple tyrosine-based signaling motifs, two SH3 domains, two putative nuclear localisation sites, and an Ena-Vasp homology (EVH1) domain binding site [2,3]. The adapter molecule SKAP55 (expressed by T cells) and the ubiquitously expressed homolog SKAP-HOM constitutively bind to ADAP. This interaction is predominantly mediated by the proline-rich region of ADAP and the SH3 domain of SKAP55/SKAP-HOM [5]. Disruption of the ADAP-SKAP55 module in T cells impairs conjugate formation between T cells and antigen-presenting cells (APCs), and lymphocyte-function-associated antigen-1 (LFA-1)-mediated adhesion [6,7]. ADAP was the first adapter molecule to be identified that couples T-cell receptor (TCR) stimulation to integrin activation and T cell adhesion (inside-out signaling) [8,9]. Recent work has established that ADAP is also required in TCR-mediated formation of the CARMA1-Bcl10-Malt1 (CBM) complex and the subsequent activation of the nuclear factor (NF)-κB pathway [10]. Furthermore, ADAP shows a phosphorylation-dependent association with the SH2 domain of the adapter molecule Nck [11,12]. Recently, a functional cooperation has been demonstrated between ADAP and Nck in stabilizing the interaction of SLP-76 and Wiskott-Aldrich syndrome protein (WASP). Thus, ADAP is also involved in the regulation of actin cytoskeleton reorganization after TCR activation [13].

Ablation of ADAP in mice does not have a major impact on the development of NK cells [14]. However, it does result in impaired positive and negative thymocyte selection, as well as inefficient population of the peripheral lymphoid organs [15]. ADAP-deficient TCR transgenic mice show a dramatically increased incidence of diabetes [16]. In a heart transplantation model, ADAP-deficient mice showed prolonged survival after a heart graft; this allograft protection was accompanied by reduced infiltration, proliferation, and activation of T cells in the allograft [17]. In another transplantation model, the rejection of intestinal allografts was ameliorated in ADAP-deficient mice [18]. These *in vivo* studies focused on the role of ADAP in T-cell function, whereas the contribution of ADAP-deficient APCs to T-cell function was not studied. To our knowledge, there have been no published reports regarding the role of ADAP in dendritic cell (DC) function.

DCs are the most efficient APCs, and they have the unique capacity to activate naïve T cells and to induce primary immune responses. They originate in the bone marrow, from where they migrate to the periphery, colonize all organs, and continually sample the surroundings for pathogens. Pathogens activate immature DCs in the peripheral organs, and after antigen uptake DCs mature into effector cells. Mature DCs lose their adhesive ability and migrate to the draining lymph node, where they present the ingested antigen to naïve T cells. In the T-cell-rich areas of the lymph node, DCs establish sequential short contacts with many T cells. This phase is followed by the establishment of long-lasting contacts and the formation of an immunological synapse that eventually results in the activation and clonal expansion of naïve T cells [19].

In this study, we analyzed the functional consequences of the loss of ADAP on dendritic cell function. We report that ADAP-deficient BMDCs show normal levels of function in antigen uptake, maturation, migration into the draining lymph nodes, antigen-specific T-cell activation, and proliferation. Importantly, however, following CD11c stimulation, the production of IL-6, TNF-α and IL-10 was diminished in ADAP-deficient BMDCs, whereas actin polymerization was enhanced. These results suggest that ADAP is required for optimal CD11c integrin-mediated DC function.

## Results

### Normal levels of skin colonization, spontaneous motility and antigen-stimulated migration are seen in ADAP-deficient BMDCs

**Figure 1:**
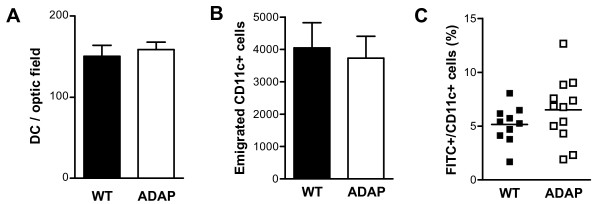
**Dendritic cell function*in vitro*: skin colonization, spontaneous emigration and antigen-stimulated migration. (A)** Mouse ear epidermal sheets from wild-type animals (WT) and ADAP- deficient animals (ADAP) were stained with an anti-MHC class II antibody and the number of MHC-II-positive dendritic cells (DC) was counted microscopically (mean + SEM, n = 8 for WT, and n = 13 for ADAP). **(B)** Skin flaps prepared from the ears of mice were floated on tissue culture medium for 48 h. The numbers of CD11c-positive cells that spontaneously migrated into the culture medium were enumerated by FACS; (mean + SEM, n = 10). **(C)** Draining lymph nodes were collected 24 h after FITC painting. Cells were stained for CD11c, and the percentages of FITC-positive and CD11c-positive cells were analyzed by FACS (n = 10).

To investigate the function of dendritic cells in ADAP-deficient mice, we first examined the distribution of DCs in the epidermis, where they persist as Langerhans cells. Ear skin explants were prepared and stained with anti-major histocompatibility complex (MHC) II antibodies, and no differences were found in the number of DCs colonizing the skin of wild-type mice or ADAP-deficient mice (Figure 1A). In addition, when the explants were cultured *in vitro*, the number of DCs that spontaneously emigrated from the epidermis was similar in ADAP-deficient mice compared with wild-type mice (Figure 1B). To assess the level of induced migration in response to dermal antigen application *in vivo*, we used the hapten fluorescein isothiocyanate (FITC) as a migration tracer. Twenty-four hours after FITC application, there was no significant difference in the percentage of FITC-positive, CD11c-positive cells in the draining lymph nodes of wild-type mice compared with ADAP-deficient mice (Figure 1C). These data suggest that ADAP does not play a major role in skin colonization by DCs. Furthermore, ADAP does not appear to be essential for the migration of DCs from the skin to the draining lymph nodes upon antigen uptake.

### ADAP-deficient BMDCs show normal levels of antigen uptake, maturation and adhesion *in vitro*

**Figure 2:**
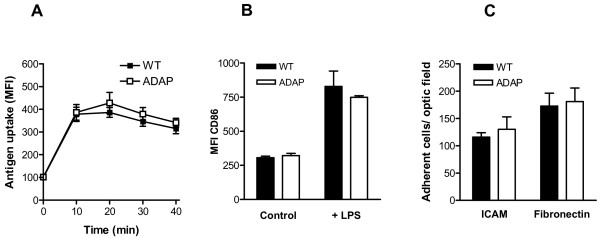
**Dendritic cell function*in vitro*: antigen uptake, maturation and adhesion. (A)** Immature BMDCs were incubated for different periods of time with DQ-OVA, and the mean fluorescence intensity (as quantification of antigen uptake) was measured by FACS (mean ± SEM, n = 6). **(B)** BMDCs were matured in the presence of LPS for 24 h. Control, immature BMDCs. The upregulation of CD86 maturation marker expression is shown as mean fluorescence intensity (MFI; mean + SEM, n = 3). **(C)** LPS-matured BMDCs were plated onto ICAM-1-coated plates and fibronectin-coated plates. After incubation for 2 h, the numbers of adherent cells were counted (mean + SEM, n = 5).

Next, we measured antigen uptake and processing by adding the fluorescent ovalbumin (OVA) analogue DQ-OVA to the culture medium of wild-type and ADAP-deficient BMDCs. BMDCs lacking ADAP and wild-type BMDCs showed no differences in these processes (Figure 2A), suggesting that ADAP is not essential for the uptake and the processing of antigens. After antigen uptake, DCs mature, lose their adhesive properties, and migrate to the regional lymph nodes. To assess the role of ADAP in these processes, we measured the upregulation of the activation markers (MHC II, CD80, CD86 and CD40) and the expression of integrins and integrin ligands (CD11c, CD18, CD29 and CD54) in LPS-matured BMDCs. The expression of maturation markers, integrins and integrin ligands was not affected by the loss of ADAP in BMDCs (Figure 2B, and data not shown). Next, we investigated the capacity of LPS-matured BMDCs to adhere to intercellular adhesion molecule 1 (ICAM-1)-coated plates. Mature ADAP-deficient DCs adhered to a level that was similar to their wild-type counterparts (Figure 2C); similar results were observed when fibronectin-coated plates were used in the adhesion assays (Figure 2C). Thus, antigen uptake, antigen processing, maturation and adhesion of BMDCs are not regulated by ADAP.

### Antigen-specific conjugate formation between ADAP-deficient BMDCs and T cells is impaired but has no impact on T-cell activation

**Figure 3:**
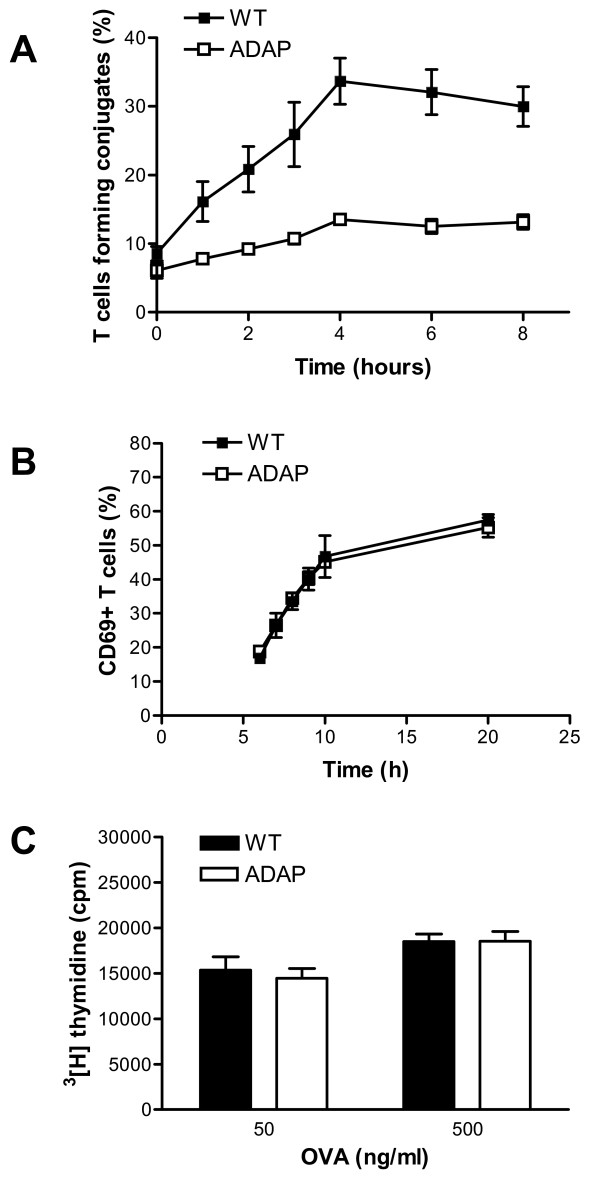
**Antigen-specific conjugate formation and T-cell activation*in vitro.*(A)** Wild-type dendritic cells (WT) or ADAP- deficient ovalbumin (OVA)-loaded mature dendritic cells (ADAP) were mixed with CFSE-loaded T cells from OT-II transgenic mice (ADAP sufficient). After co-incubation, conjugates were fixed by the addition of 1 % paraformaldehyde at different time points. The significant difference between the curves (*P* < 0.01) was assessed by statistical analysis using two-way ANOVA. The plots show the mean values of five independent experiments. **(B)** In the same experimental set-up described in (A), cells were harvested at the indicated time points, stained with anti-CD69 antibodies, and the numbers were measured by FACS. **(C)** BMDCs were matured in the presence of LPS and loaded with OVA, and mixed with T cells from OT-II transgenic mice in a ratio of 1:10. T-cell proliferation was evaluated after 3 days by measuring [^3^ H]thymidine incorporation (mean + SEM, n = 9).

The activation of naïve T lymphocytes requires prolonged interaction of DCs with T cells [19]. To analyze the capacity of ADAP-deficient and wild-type BMDCs to form antigen-specific conjugates with T cells *in vitro*, we labeled OT-II TCR transgenic T cells (ADAP sufficient) with carboxyfluorescein succinimidyl ester (CFSE) and co-incubated them with wild-type or ADAP-deficient LPS-matured BMDCs loaded with OVA_353-363_. As shown in Figure 3A, the formation of antigen-specific conjugates, measured by fluorescence-activated cell sorting (FACS), was almost completely abrogated when ADAP-deficient DCs were used. To assess the possible impact of the abrogated conjugate formation on T-cell activation, we measured CD69 upregulation (Figure 3B) and T cell proliferation *in vitro* (Figure 3C). Despite this strongly impaired conjugate formation, neither the expression of the early activation marker CD69, nor the incorporation of ^3^ H]thymidine in T cells as a marker of DNA synthesis, seemed to be affected by the loss of ADAP expression in DCs.

**Figure 4:**
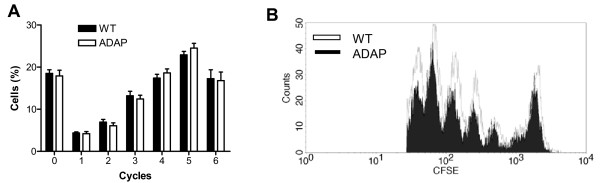
**Antigen-specific T-cell proliferation*in vivo*. (A)** Transgenic OT-II T cells were loaded with CFSE and adoptively transferred into wild-type mice. After 24 h, wild-type BMDCs (WT) and ADAP-deficient BMDCs (ADAP), which had been preloaded overnight with ovalbumin in the presence of LPS, were injected subcutaneously into wild-type mice. After another 72 h, draining lymph nodes were harvested, and T-cell proliferation was evaluated by FACS. The percentage of cells undergoing 0 to 6 cycles of division is shown (mean + SEM, n = 6). **(B)** A representative profile of the CFSE dilution.

We next asked the question whether the diminished conjugate formation between antigen-specific T cells and ADAP-deficient BMDCs would have consequences for immune activation *in vivo*. To assess this, we investigated antigen-specific T-cell proliferation *in vivo* by adoptive transfer of CFSE-labeled OT-II transgenic T cells into wild-type recipient mice. After 24 h, we injected the mice subcutaneously either with ADAP-sufficient or with ADAP-deficient BMDCs pulsed with OVA in the presence of LPS. The CFSE dilution profile in Figure 4A shows that ADAP-deficient BMDCs supported OT-II T-cell proliferation to the same level of efficiency as that seen in wild-type BMDCs. Similarly, ADAP-deficient BMDCs and wild-type BMDCs induced a comparable strong proliferation of transgenic OT-II T cells, undergoing up to six cell cycles (Figure 4B). Thus, despite an impaired conjugate formation with T cells, ADAP-deficient DCs were able to fully activate T cells *in vitro* and *in vivo*.

### ADAP-deficient BMDCs produce normal levels of cytokines and show normal levels of TLR4 signalling

**Figure 5:**
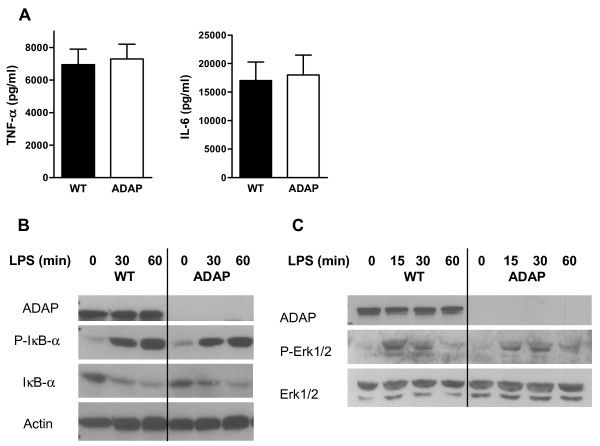
**Cytokine production and signaling after LPS stimulation. (A)** BMDCs from wild-type animals (WT) and ADAP-deficient animals (ADAP) were stimulated for 24 h in the presence of LPS, and the production of cytokines TNF-∝ and IL-6 was measured in the supernatants (mean + SEM, n = 7 to 9). BMDCs were stimulated with LPS for the indicated time, lysed, and then immunoblotted with antibodies to investigate NF-κB signaling **(B),** and the MAP kinase signaling pathway **(C)**. One representative of three independent experiments is shown.

DCs are main producers of cytokines, which play a major role in the induction of an immune response. Next, we measured the secretion of pro-inflammatory cytokines in the supernatants of ADAP-sufficient and ADAP-deficient BMDCs after stimulation with LPS for 24 h *in vitro*. The cytokines TNF-α and IL-6 were produced in large amounts, and no differences were detected between the amounts produced by wild-type and ADAP-deficient DCs (6,953 ± 942 and 7,292 ± 919 pg/ml TNF-∝, and 17,021 ± 3,257 and 18,001 ± 3,466 pg/ml IL-6, for wild-type and ADAP-deficient BMDs, respectively; Figure 5A). These results suggest that ADAP is not involved in the production of TNF-∝ and IL-6 after stimulation of BMDCs by LPS.

LPS binding to the TLR4 receptor complex activates the transcription factor NF-κB and, in addition, activates mitogen-activated protein (MAP) kinases. Therefore, we used western blotting to investigate the phosphorylation and subsequent degradation of IκB-α as a main component of the NF-κB signaling pathway. As shown in Figure 5B, levels of phosphorylation and degradation of IκB-∝ after LPS stimulation were found to be similar in wild-type and ADAP-deficient BMDCs. Furthermore, ADAP-deficient and wild-type BMDCs showed similar levels of activation of the MAP kinase Erk1/2 (Figure 5C), indicating that ADAP is not involved in the signaling cascades after TLR4 activation by LPS.

### ADAP-deficient BMDCs show defects in CD11c-mediated responses

**Figure 6:**
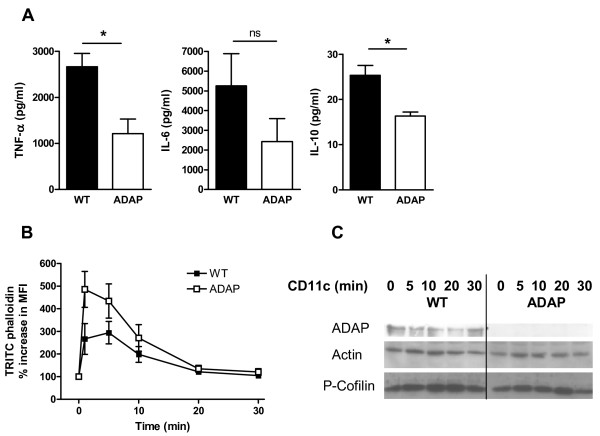
**Cytokine production and actin polymerization after CD11c stimulation. (A)** Production of cytokines TNF- ∝, IL-6 and IL-10 by BMDCs of wild-type animals (WT) and ADAP-deficient animals (ADAP) was measured in the supernatants 24 h after stimulation with anti-CD11c (mean + SEM, n = 3, *P < 0.05, ns = non significant). **(B)** BMDCs of WT and ADAP were incubated with anti-CD11c. After incubation, cells were permeabilized and stained with TRITC-phalloidin. The cellular F-actin content was analyzed by FACS. Results are expressed as the percentage increase in mean fluorescence intensity (mean ± SEM, n = 6; *P* = 0.0031 for the two curves, as assessed by two-way ANOVA). **(C)** BMDCs of WT and ADAP were stimulated with anti-CD11c for 0, 5, 10, 20, and 30 min. Lysates were separated by SDS-PAGE, and immunoblotted with the indicated antibodies. One representative of three independent experiments is shown.

In addition to its role in inside-out signaling, ADAP is implicated in integrin-mediated outside-in signaling in T cells [20]. To assess the role of ADAP in outside-in signaling in BMDCs, we first investigated cytokine production following CD11c stimulation. After stimulation with anti-CD11c and in the absence of additional stimuli, the production of IL-6, TNF-∝ and IL-10 was clearly reduced in ADAP-deficient BMDCs compared with that in wild-type BMDCs (2,665 ± 292 and 1,209 ± 322 pg/ml TNF-∝, 5,257 ± 1,627 and 2,428 ± 1,167 pg/ml IL-6, and 25.3 ± 2.2 and 16.3 ± 0.9 pg/ml IL-10, for wild-type and ADAP-deficient BMDCs, respectively; Figure 6A).

Integrin-mediated outside-in signaling leads to rearrangements of the actin cytoskeleton which can be estimated by measuring the amount of actin that polymerizes and binds to phalloidin. ADAP-deficient BMDCs showed a significantly higher increase in actin polymerization than that in their wild-type counterparts (Figure 6B). Given the effect on F-actin, we assessed CD11c-mediated activation of the actin-severing protein cofilin, which is an essential component of the machinery that regenerates actin filaments. Cofilin is inactivated by phosphorylation. After 5 and 10 min of CD11c stimulation, ADAP-deficient BMDCs showed lower amounts of phosphorylated (inactive) cofilin compared with wild-type BMDCs (Figure 6C). Thus, CD11c stimulation of ADAP-deficient BMDCs resulted in enhanced actin polymerization that was accompanied by the presence of more active cofilin. These results indicate that ADAP is involved in actin polymerization and cytokine production upon CD11c-mediated outside-in signaling.

## Discussion

In this study, we investigated the consequences of the loss of ADAP on the function of BMDCs. We found that: (i) most DC functions, such as antigen uptake, adhesion, migration, maturation and T-cell activation, were not affected by the loss of ADAP; (ii) ADAP was not involved in the TLR4 signaling pathway after LPS stimulation; (iii) ADAP-deficient BMDCs showed defects in CD11c-stimulated cytokine production, and displayed enhanced CD11c-mediated actin polymerization.

ADAP has a homolog that is expressed during normal myelopoiesis: PML-RAR alpha-regulated adapter molecule 1 (PRAM-1). PRAM-1 was originally identified in promyelocytic leukemia cells upon all-*trans* retinoic acid-induced granulocyte differentiation. PRAM-1 shares structural homologies with ADAP, and has been shown to interact with SLP-76, SKAP-HOM and the Src family kinase Lyn in myeloid cells [21]. A previous analysis of PRAM-1-deficient neutrophils exhibit defects in adhesion-dependent reactive oxygen species (ROS) production and degranulation [22]. The function of PRAM-1-deficient DCs has not yet been investigated. Thus, one possible explanation for the predominantly normal function of ADAP-deficient BMDCs is compensation by PRAM-1. To address a possible functional redundancy between ADAP and PRAM-1 in DCs, ADAP/PRAM-1 double-deficient mice should be investigated.

In contrast to the almost normal DC function, we found impaired formation of antigen-specific conjugates between ADAP-deficient BMDCs and T cells. Surprisingly however, the antigen-specific T-cell activation and proliferation were found to be normal *in vitro* and *in vivo*. The most likely explanation for this observation is that measurement of stable conjugates *in vitro* might not reflect the physiological situation of T-cell activation. This idea is supported by the serial encounter model of T-cell activation. This model proposes a dynamic process of sequential migratory contacts of T cells with different DCs resulting in summation of signals finally resulting in full T-cell activation [23]. This might explain our observation of normal T-cell activation by ADAP-deficient BMDCs irrespective of impaired stable conjugate formation. Furthermore, DCs actively rearrange their cytoskeleton during the antigen-specific interaction with T cells [24]. The increased actin polymerization of ADAP-deficient BMDCs could counterbalance the impaired conjugated formation leading to normal T cell activation. Studies imaging cell morphology, conjugate formation, and actin polymerization using microscopy will elucidate this question.

In our previous study we reported that the absence of SKAP-HOM in BMDCs resulted in delayed conjugate formation with antigen-specific T cells. Furthermore, SKAP-HOM-deficient DCs were less effective in inducing antigen-specific T cell proliferation in vivo [25]. This result is different from that observed in ADAP-deficient DCs. We describe here impaired antigen-specific conjugate formation but normal T-cell activation. These data suggest that some ADAP functions do not depend on SKAP-HOM and vice versa. This hypothesis was corroborated by data collected in T cells showing two functionally distinct pools of ADAP: the ADAP/SKAP55 complex is required for TCR-mediated integrin activation, whereas the TCR-mediated NF-κB activation involves ADAP but not SKAP55 [26]. The proof of existence of distinct pools of ADAP in DCs should be addressed in future studies.

TLR4 serves as the main LPS-binding component and initiates signal transduction by recruiting the intracellular adapter molecules TIRAP and MyD88. MyD88 is a signaling component used by nearly all TLRs, and it activates the transcription factor NF-κB and the MAP kinase pathway, thereby inducing the production of proinflammatory cytokines, including IL-6 and TNF-∝ [27]. The kinase TAK1 is another component involved in the signaling pathways downstream of TLR4 [28]. ADAP has been shown to be involved in the regulation of NF-κB activation in T cells via an association with CARMA1 [10]. More recently, it has been reported that ADAP associates with TAK1, and that this interaction is critical for the recruitment of TAK1 to the CBM complex in T cells [29]. Our results shown here clearly demonstrate that ADAP is not involved in the activation of NF-κB and MAP kinases after TLR4 stimulation of BMDCs with LPS.

In contrast to the well-established immunoreceptor and TLR signaling pathways, the signaling events after external triggering of integrins (outside-in signaling) remain poorly understood. Recent reports have shown that the association between SLP-76 and ADAP is critical for downstream signaling of integrins in T cells [30]. In addition, LFA-1 stimulation promotes association of ADAP with cytoskeletal structures (‘actin clouds’), which facilitate T cell adhesion [31]. Further, ADAP is required for LFA-1-induced T-cell polarization, T-cell motility and F-actin clustering [20]. Those reports collectively demonstrate that ADAP is involved in integrin outside-in signaling in T cells. ADAP-deficient neutrophils display reduced adhesion-dependent ROS production [32]. In addition, it has been shown that SLP-76-deficient BMDCs exhibit impaired adhesion and altered patterns of actin assembly and podosome distribution following integrin ligation [33]. Given the association of SLP-76 and ADAP, one could hypothesize that ADAP is involved in integrin outside-in signaling in DCs as well. Indeed, we demonstrate here that ADAP-deficient BMDCs show impaired production of IL-6, TNF-∝ and IL-10 after CD11c triggering. Although the precise mechanism of reduced cytokine production remains to be elucidated, the involvement of ADAP in CD11c-mediated cellular responses seems to be the most likely explanation. Recent reports indicate that integrins, which do not share structural similarity to classical immunoreceptors, also signal by an ITAM-based immunoreceptor-like mechanism in myeloid cells [34]. This integrin signaling, through common pathways utilized by immunoreceptors, could provide a mechanism by which leukocyte adhesion can regulate activation of cellular responses such as ROS production, degranulation, cytokine secretion [35].

CD11c (integrin ∝X β2; p150/95; complement receptor CR4) serves as marker for DCs, although its function in DC biology remains unclear. The limited numbers of known ligands include fibrinogen, ICAM-1/2 and iC3b [36]. The impaired production of IL-6, TNF-α and IL-10 after CD11c triggering might have consequences on the induction of an adaptive immune response in ADAP-deficient mice. It would be interesting to test this hypothesis using *Listeria monocytogenes* infection, an infection model clearly depending on DCs [37,38].

Integrin activation is accompanied by reorganization of the actin cytoskeleton, leading to cell adhesion. Here, we describe an increased F-actin content in ADAP-deficient BMDCs after CD11c triggering. This increased actin polymerization was accompanied by enhanced dephosphorylation of the small actin-binding protein cofilin, a critical step in the reorganization of actin filaments. Hence, ADAP appears to negatively regulate actin polymerization in DCs. In line with this hypothesis is our previous study where we demonstrated that the loss of the adapter protein SKAP-HOM also results in enhanced actin polymerization after CD11c triggering of BMDCs [25]. Given the direct interaction of ADAP and SKAP-HOM, these results suggest that the molecular complex of both molecules is involved in CD11c-triggered outside-in signaling leading to actin polymerization. Future studies will dissect the mechanism how ADAP and its binding partners regulate CD11c-mediated outside-in signaling.

## Conclusions

In summary, we provide evidence that ADAP is involved in outside-in integrin signaling in DCs after triggering of CD11c. This integrin signaling provides a mechanism by which DCs can regulate cellular responses leading to decreased cytokine production and enhanced actin polymerization in the absence of ADAP. Our findings have important implications for the understanding of the role of ADAP in CD11c-triggered outside-in signaling in DCs.

## Materials and methods

### Mice

ADAP-deficient mice [8] were backcrossed to the C57BL/6JBom strain for at least ten generations. Transgenic OT-II mice expressing the TCR specific for chicken OVA_323-339_ were purchased from The Jackson Laboratory. Mice were bred and maintained under specific-pathogen-free conditions in the central animal facility of the Medical Faculty of the University of Magdeburg. In all experiments, 8- to 12-week-old littermate mice were used. All procedures were conducted according to protocols approved by the local authorities.

### Antibodies and flow cytometry

Antibody against MHC II (clone 2 G9) was kindly provided by M Leverkus (Mannheim). All other antibodies used for flow cytometry were from BD Biosciences. Flow cytometry was performed on a FACSCalibur using CellquestPro software (Becton Dickinson).

### Production of BMDCs

Production of BMDCs was performed as previously described [25]. Briefly, bone marrow cells (10^6^ cells/ml) were incubated in RPMI 1640 medium supplemented with 10 % fetal calf serum (FCS), 100 U/ml penicillin, 100 μg/ml streptomycin (all Biochrom AG) and 50 μM β-mercaptoethanol (the medium is henceforth referred to as complete medium) with the addition of 20 ng/ml rGM-CSF (PeproTech, Hamburg, Germany). On day 3, fresh medium was added (3.5 volumes). On day 5, cells in the supernatant were centrifuged, resuspended in fresh complete medium, and added back to the culture. On day 7, immature DCs were harvested, washed twice, and either used directly or matured in the presence of 100 ng/ml LPS from *Salmonella minnesota* (Sigma) for an additional 24 h. Maturation was routinely tested by MHC II and CD86 upregulation. Purity was >90 %, as checked by flow cytometry using CD11c staining.

### Epidermal sheet preparation and skin DC emigration

The ears of the mice were split into dorsal and ventral halves, and the dorsal half was floated split-side down in 1 ml complete medium for 2 days. Cells that had migrated were resuspended, stained for CD11c, and measured by FACS. Alternatively, the dorsal half was incubated in ammonium thiocyanate (0.5 M) for 20 min at room temperature. Next, the epidermis was separated from the dermis and fixed in acetone for 20 min at room temperature. After rehydration in PBS, sheets were permeabilized in 0.1 % saponin, blocked with 0.5 % BSA, and incubated with 2 G9 antibodies for 2 h. After two washing steps, sheets were incubated with FITC-labeled secondary antibodies (Dianova, Hamburg, Germany) for 2 h.

### *In vivo* migration (FITC painting)

One side of the clipped ventral abdomen of the mice was stained with 300 μl FITC (5 mg/ml dissolved in dibutylphtalate:acetone, 1:1 volume:volume). As a control, the other side of the abdomen was stained with solvent only. After 24 h, mice were killed and cells of the draining lymph nodes were collected and stained for CD11c. The percentage of CD11c-positive FITC-positive cells was determined by FACS.

### Endocytosis assay and antigen processing

Immature DCs were washed and resuspended in PBS at a density of 1 × 10^6^ cells/450 μl, and DQ-OVA (Invitrogen, Karlsruhe, Germany) was added to a final concentration of 100 μg/ml. Cells were incubated for 15 min at 37 °C. The uptake of DQ-OVA was terminated by the addition of ice-cold PBS supplemented with 10 % FCS. The cells were then washed twice with ice-cold PBS. To assess antigen processing, cells were incubated further at 37 °C, and then washed and resuspended in cold PBS. Antigen uptake and processing were evaluated by FACS.

### Cell adhesion assay

Microtiter plates (24 well; Costar) were coated with 200 μl fibronectin/well (100 μg/ml; Roche Diagnostics) for 16 h at 4 °C, washed three times with PBS and blocked with 1 % BSA in PBS for an additional 2 h. Coating of the plates with murine ICAM-1 was performed as described [39]. BMDCs (5 × 10^5^ cells) were resuspended in 500 μl RPMI and incubated at 37 °C for 2 h. Non-adherent cells were washed off, and adherent cells were counted in four different optic fields.

### Conjugate formation, CD69 upregulation and T-cell proliferation assay *in vitro*

Immature DCs were matured by the addition of 100 ng/ml LPS in the presence of 1 μg/ml OVA peptide (OVA_323-339_). After overnight incubation, mDCs were washed and resuspended at a concentration of 1 × 10^6^ cells/ml in complete medium. T cells were purified from OT-II transgenic mice (bearing the TCR specific for OVA_323-339_) by negative selection via magnetic separation using AutoMACS. Isolated T cells were labeled with CFSE (0.5 μM) at 37 °C for 10 min. After washing, labeled T cells were resuspended at a concentration of 1 × 10^6^ cells/ml in complete medium. Peptide-loaded mDCs (5 × 10^4^ cells/100 μl) were mixed with T cells (5 × 10^4^ cells/100 μl) in a 96-well plate (ratio 1:1). After co-incubation for the indicated time, cells were fixed by adding 200 μl 4 % paraformaldehyde. After 20 min at 37 °C, conjugate formation was measured by FACS. CFSE-positive events with increased forward scatter were considered to be conjugates. Alternatively, cells were stained for CD4 and CD69. For T cell proliferation, 1 × 10^3^ mDCs were used to stimulate 1 × 10^4^ T cells (ratio 1:10). Cells were co-incubated for 72 h at 37 °C, and 0.5 μCi [^3^ H]thymidine was added for the last 8 h. The incorporated radioactivity was measured by liquid scintillation counting (1450 MicroBeta Trilux; PerkinElmer Wallac GmbH).

### Antigen-specific T-cell proliferation *in vivo*

Purified OT-II T cells were labeled with CFSE (5 μM) and 2 × 10^6^ cells were injected intravenously into wild-type mice. BMDCs were incubated overnight in the presence of 100 ng/ml LPS and 5 μg/ml OVA. After extensive washing, DCs were resuspended at a concentration of 1 × 10^7^ cells/ml, and a 50 μl volume of cell suspension was injected subcutaneously 1 day later. After 3 days, the draining lymph nodes were isolated, and the extent of transgenic T-cell proliferation was assessed by CFSE dilution profile using flow cytometry.

### Cytokine concentration

Levels of cytokines in culture supernatants were determined by using the inflammatory cytometric bead array kit (Becton Dickinson).

### Western blot analysis

Immature DCs (2×10^6^ cells) were stimulated with LPS (100 ng/ml) at 37 °C. For CD11c stimulation, BMDCs were left unstimulated or were preincubated with anti-CD11c on ice for 5 min before subsequent incubation for 0, 30 or 60 min at 37 °C. The stimulation was stopped by adding ice-cold PBS. The lysates were prepared as described previously [25,39]. The following primary antibodies were used: anti-ADAP (FYB/SLAP-130; BD Biosciences), anti-IKB-∝, anti-phospho-IkB-∝, anti-phospo-cofilin (all Santa Cruz Biotechnology Inc.), and anti-phospho-Erk1/2, anti-ERK1/2 and anti-β-actin (all Cell Signaling Technology, Inc.). Specific binding was detected using peroxidase-conjugated secondary antibodies by applying the enhanced chemiluminescence system (GE Healthcare), according to the manufacturer’s protocol.

### Actin polymerization

BMDCs (2×10^6^ cells/ml in PBS) were stimulated with 1μg/ml affinity-purified Armenian hamster anti-mouse CD11c (BD Biosciences). BMDCs were preincubated with anti-CD11c on ice for 5min before subsequent stimulation at 37 °C. At the end of the stimulation time, 4 % formaldehyde, 0.2% saponin and TRITC-phalloidin (5 μg/ml) were added. The tubes were vortexed and kept at room temperature for 15 min. After washing, cells were resuspended 1% paraformaldehyde, and actin polymerization was measured by flow cytometry. Results are expressed as percent increase in mean fluorescence intensity.

### Statistical analysis

Values are expressed as mean ± SEM of at least three independent experiments, unless otherwise indicated, where “n” represents the number of mice. ANOVA was used to assess the statistical significance of the differences. Probability values of *P*<0.05 were considered significant.

## Abbreviations

ADAP: Adhesion and degranulation promoting adapter protein; APC: Antigen-presenting cell; BMDC: Bone-marrow-derived dendritic cell; CBM: CARMA1-Bcl10-Malt1; CFSE: Carboxyfluorescein succinimidyl ester; DC: Dendritic cell; FITC: Fluorescein isothiocyanate; FCS: Fetal calf serum; ICAM-1: Intercellular adhesion molecule 1; IL: Interleukin; ITAM: Immunoreceptor tyrosine-based activation motif; LFA-1: Lymphocyte-function-associated antigen-1; LPS: Lipopolysaccharide; MHC: Major histocompatibility complex; NF: Nuclear factor; OVA: Ovalbumin; TCR: T-cell receptor; TNF: Tumor necrosis factor.

## Competing interests

The authors declare that they have not competing interests.

## Authors’ contributions

MT and AR contributed to the design of the study. MT, SE and AR performed the acquisition and analysis of data. AR and SE contributed to the writing of this manuscript. DR participated in the conception of the study and helped to draft the manuscript. BS was involved in drafting and revising the manuscript. All authors read and approved the final version of this manuscript.
